# Wildfires modify the parasite loads of invasive cane toads

**DOI:** 10.1098/rsbl.2021.0470

**Published:** 2021-12-22

**Authors:** Shannon W. Kaiser, Matthew J. Greenlees, Richard Shine

**Affiliations:** Department of Biological Sciences, Macquarie University, NSW 2109, Australia

**Keywords:** *Bufo marinus*, habitat change, host–parasite, invasive species

## Abstract

The frequency and severity of wildfires are increasing due to anthropogenic modifications to habitats and to climate. Post-fire landscapes may advantage invasive species via multiple mechanisms, including changes to host–parasite interactions. We surveyed the incidence of endoparasitic lungworms (*Rhabdias pseudosphaerocephala*) in invasive cane toads (*Rhinella marina*) in near-coastal sites of eastern Australia, a year after extensive fires in this region. Both the prevalence of infection and number of worms in infected toads increased with toad body size in unburned areas. By contrast, parasite load decreased with toad body size in burned areas. By killing moisture-dependent free-living lungworm larvae, the intense fires may have liberated adult cane toads from a parasite that can substantially reduce the viability of its host. Smaller toads, which are restricted to moist environments, did not receive this benefit from fires.

## 1. Introduction

Over recent decades, changes to temperature and rainfall patterns are causing more extreme events such as bushfire, flood and drought [1,2]. Those phenomena can disrupt natural systems in many ways, some of them obvious (such as direct mortality and habitat destruction) but others less apparent (such as the disruption of important interspecific interactions). For example, the open landscapes produced by fire can facilitate predation upon individuals of prey species that survived the fire [3], and herbivores may migrate to post-burn areas to exploit the newly available nutrient-rich vegetation [4]. Many invasive species are major beneficiaries of the habitat changes, able to thrive in disturbed habitats [5].

One important but under-studied impact of intense fires involves changes to parasite load. If a parasitic species includes a free-living stage in the life cycle, high temperatures and ground-cover elimination by fire may substantially reduce parasite abundance and hence reduce deleterious effects of parasites on their hosts (e.g. [6]). Thus, for example, wildfires have been reported to reduce ectoparasitic mite infestation rates in lizards [7] and chytrid fungus loads of boreal toads [8]. The impacts of fires on parasite loads of invasive species do not appear to have been studied, however.

In the summer of 2019–20, a prolonged drought followed by atypically high summer temperatures resulted in massive fires across near-coastal regions of eastern Australia [9]. The fires burnt almost 100 000 km^2^ of vegetation [9] in an area that was already impacted by habitat destruction, disease, drought and invasive species [10]. At first sight, we might expect an intense fire to be deleterious to an invasive amphibian such as the cane toad (*Rhinella marina*), by killing toads and eliminating the moist refuges required by this water-dependent species [11]. However, cane toads actively prefer relatively open habitats for foraging [12] and thus can thrive in post-fire landscapes [13]. Plausibly, intense fire also may reduce the incidence of parasitic lungworms that are widespread in Australian cane toads [14] and can enforce major reductions in host viability [15]. To test this idea, we compared parasite loads in cane toads from adjacent sites that differed in whether or not they had been burned during the 2019–2020 bushfires.

## 2. Material and methods

### (a) Study species

Cane toads (*Rhinella marina*; previously *Bufo marinus*) are large (exceptionally, to greater than 1 kg) bufonid anurans native to northeastern South America but translocated to many countries worldwide in the 1930s for pest control in commercial agriculture [12]. Adult cane toads are primarily nocturnal (but see [16]), foraging at night for insect prey and retreating to moist shelter-sites by day [17]. Small toads are restricted to the margins of natal ponds until they are large enough to withstand desiccating conditions [18], but adult toads are highly dispersive and can use arid habitats if moist diurnal retreat-sites are available (e.g. [19]).

The nematode lungworm *Rhabdias pseudosphaerocephala* is abundant and widespread in cane toads across Australia as well as in the species' native range, having been brought to Australia when the toads were introduced to that continent in 1935 [20]. Adult worms are hermaphroditic and live in the lining of the host lung, producing larvae that are defaecated by the host [21]. Infective larvae are free-living in moist soil and enter the bodies of new hosts by crawling through areas of thin epidermis, such as around the eyes and cloaca [22]. Experimental manipulation of parasite loads in free-ranging toads has revealed strongly negative effects on lungworms on host survival and behaviour [15]. Surveys in tropical Australia showed that prevalence and intensity of lungworm infections in cane toads increase with toad body size and vary seasonally [14].

### (b) Methods

We obtained 512 cane toads that had been collected by community environmental groups (Border Ranges Richmond Valley LandCare Network (BRRVLN) and Clarence Valley Landcare) and another 62 animals in the course of our own fieldwork. All toads were humanely euthanized by cooling then freezing [23]. We asked the LandCare groups to record the location and date of collection, and whether the area was burned or unburned by the 2019–2020 fires (based on maps provided by the Rural Fire Service, and checked by onsite inspections). All toads came from northeastern New South Wales (NSW), within 75 km of the city of Casino (see electronic supplementary materials for locations). Collections were made in November 2020 to April 2021, 11 to 16 months since intense wildfires affected this region in December 2019 and January 2020.

**Figure 1.**
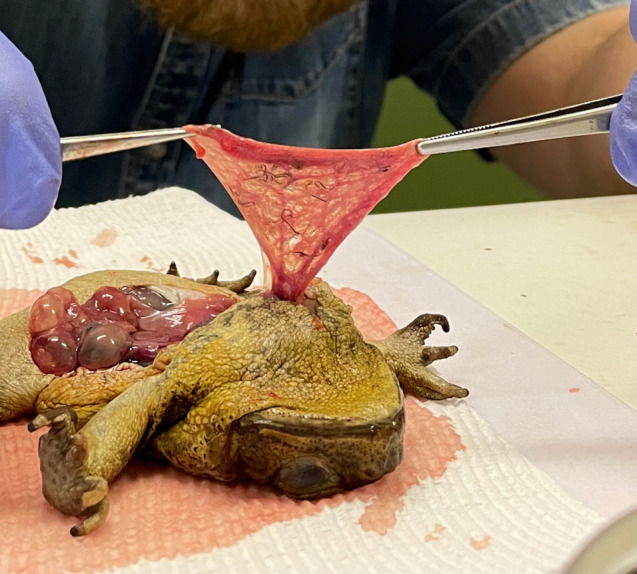
A dissected cane toad (*Rhinella marina*), showing lungworms (*Rhabdias pseudosphaerocephala*) in the left lung of the toad. Photograph by Matthew Greenlees.

We thawed 572 toads and recorded their body length using Vernier callipers (snout urostyle length, =SUL) and sex, then dissected them. To score parasite loads, we made a midventral incision, stretched the left lung out of the body with forceps and counted lungworms (figure 1). From those data, we quantified parasite load in terms of *prevalence* (proportion of toads that contained visible lungworms) and *abundance* (number of lungworms per infected toad, omitting non-infected hosts).

### (c) Statistical analysis

Data analysis was conducted in R v. 4.0.0 [24] using the packages ‘lme4’ [25] for mixed-effects models, ‘nlme’ [26] for linear models and ‘tidyverse’ [27] for data manipulation. We analysed data on parasite prevalence using a mixed model with a binary distribution and logit link function. For data on abundance, we used a mixed model with negative binomial link distribution and log link. In both cases, we included fire condition (burned versus unburned) as a factor, and toad body size (SUL) as a covariate, plus the interaction between these two variables. Population (sampling location) was included as a random factor. Preliminary analyses showed no significant differences in parasite load between sexes, nor any interaction between sex and body size, so we report only analyses on the combined dataset.

Because initial analyses identified a strong interaction between fire condition and body size in driving parasite load, we arbitrarily divided toads into juvenile versus adult animals (i.e. greater or less than 70 mm SUL, as toads below this size were difficult to sex). We then repeated the above analyses with fire condition as the factor (plus population as a random variable), to see if fire condition significantly affected parasite loads in either juvenile toads or in their adult conspecifics.

To explore possible shifts through time, we added ‘number of months since fire’ as an additional covariate in the analyses above.

## 3. Results

### (a) Sample sizes and mean body sizes of toads

We obtained data on parasite loads of 572 toads (471 adults, 101 juveniles): 279 from five burned sites and 293 from five unburned sites. Of those 572 toads, 35% contained lungworms in the left lung (range 0 to 83 worms per toad). Mean body sizes of toads did not differ significantly between burned and unburned sites (*t* = −0.93, d.f. = 8, *p* = 0.38).

### (b) Effects of body size and fire condition on parasite load

**Figure 2.**
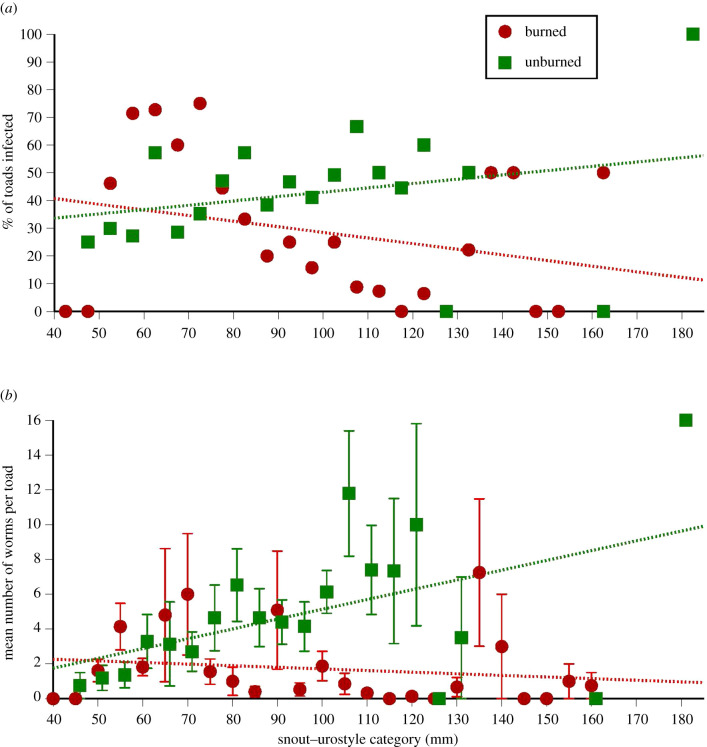
The relationship between a cane toad's body size (snout–urostyle length) and its parasite load in burned and unburned sites; (*a*) shows data for prevalence (proportion of toads infected with lungworms) whereas (*b*) shows data for abundance (number of lungworms per toad, omitting data for non-infected individuals). To clarify patterns in the data, the figure shows data (means and associated standard errors) for each 5 mm SUL size class of toads. However, statistical analyses in this paper use body size as a continuous rather than categorical variable.

Analyses of parasite prevalence and abundance yielded similar patterns and conclusions (figure 2*a,b*). Parasite loads tended to be higher in unburned sites, with a strong effect of toad body size. In unburned sites, larger toads were more likely to be infected and contained more lungworms. By contrast, larger toads in burned sites were less likely to be infected and contained fewer worms (figure 2*a*,*b*). As a result, the interaction term between toad body size and fire condition was significant both for parasite prevalence (*Z* = 2.87, d.f. = 567, *p* < 0.005) and abundance (*Z* = 2.47, d.f. = 187, *p* < 0.014).

The data in figure 2 suggest that parasite loads of small toads were unaffected by fire condition, whereas larger toads benefited considerably. When we divided toads into juveniles and adults (i.e. greater or less than 70 mm SUL), and repeated the analyses with fire condition as the only factor (plus population as a random variable), fire condition did not affect parasite loads in juveniles (*t* = 0.225, d.f. = 6, *p* = 0.83) but did so in adults (*t* = −2.75, d.f. = 8, *p* = 0.025).

In analyses with the additional covariate ‘number of months since fire’, parasite loads decreased over time but did so equally in burned and unburned sites, consistent with a seasonal effect rather than an impact of fire *per se*. With time since fire included as a covariate, fire condition continued to significantly affect parasite loads (prevalence, *p* < 0.0001; abundance, *p* < 0.012).

## 4. Discussion

Intense wildfires affected the parasite loads of cane toads that were collected a year post-fire, but that effect depended upon the body size of the host. The parasite loads of small toads were unaffected by fire, whereas those of large toads were lower in burned areas (figure 2*a*,*b*). A toad's sex did not affect its parasite load, consistent with earlier studies [14,22]. The pattern for parasite load to increase with toad body size, as seen in our unburned sites, mirrors the results of an earlier study in tropical Australia [14], whereas a broader-scale sampling study reported that parasite loads were highest in toads of intermediate body sizes [28]. The effect of fire on the allometry of parasite load—the main result of our analyses above—suggests that this allometry may depend upon local habitats.

Why should a toad's body size affect its parasite load? Several reasons may explain why parasite load might be expected to increase in larger toads (as observed in a previous study [14] and in unburned sites in our own work). For example, larger toads are likely to be older, thus, have had a longer period of exposure to parasites, and may have less effective immune responses [29]; they may also be more vulnerable simply because they offer a larger target for uptake of parasite larvae [14].

The fire dependency of this size effect in our data suggests an additional hypothesis. Larger toads are more resistant to desiccation and thus tend to use drier microhabitats (e.g. [17]). Such an ontogenetic shift in habitat use might exacerbate exposure to parasites if toads in dry sites cluster in the few available shelters, increasing opportunities for parasite transmission (as may explain seasonal variation in parasite loads of tropical toads [14]). Alternatively, in an area with relatively low densities of toads (and thus, little sharing of refuge sites), drier areas may curtail the longevity of free-living infective larvae [22] and thus reduce the infection rate. The latter situation may apply to post-fire sites in our own results.

Our study does not identify the proximate mechanism by which a previous fire (a year earlier) reduced parasite loads in adult cane toads. One possibility is that infective larvae in sites away from water were killed by fire. Another is that the open sun-exposed landscape post-fire provided few places for larvae to survive, except around the margins of ponds. Experimental studies have confirmed that lungworm larvae are unable to survive in dry soil [30]. Yet another is that the toads currently found in the broader landscape (i.e. away from water) within burned areas are recent immigrants from nearby sites. If lungworm infection reduces dispersal rates of toads [15] (but see [31]), such immigrants might be less heavily infected than most of their conspecifics. Future work could test these possibilities by following the time course of toad colonization of post-fire habitats at a finer temporal scale, and by directly measuring rates of survival of lungworm larvae, and of infection of toads, in the refuge sites available in unburned versus burned locations. More generally, given that parasitism can massively influence the viability of their hosts, we need additional research on the ways in which anthropogenically driven shifts in phenomena such as droughts, floods and bushfires modify important interspecific interactions including predation, competition and parasitism. Invasive species tend to thrive in highly modified habitats, for multiple reasons (e.g. [3,12]), and our study suggests that abiotic shifts caused by intense fire may contribute to that success, by releasing invaders from the negative effects of parasites.

## Data accessibility

All R code is included as supplementary files. Data on collection dates and locations, toad body sizes and parasite loads are available from Dryad https://doi.org/10.5061/dryad.rxwdbrv9b. The data are provided in the electronic supplementary material [32].
